# Computed Tomography Angiography for the Diagnosis of Coronary Artery Disease Among Patients Undergoing Transcatheter Aortic Valve Implantation

**DOI:** 10.1007/s12265-021-10099-8

**Published:** 2021-02-04

**Authors:** David Meier, Arnaud Depierre, Antoine Topolsky, Christan Roguelov, Marion Dupré, Vladimir Rubimbura, Eric Eeckhout, Salah Dine Qanadli, Olivier Muller, Thabo Mahendiran, David Rotzinger, Stephane Fournier

**Affiliations:** 1 Department of Cardiology, Lausanne University Hospital, Rue du Bugnon 46, 1011 Lausanne, Switzerland; 2 Faculty of Biology and Medicine, Lausanne University, Lausanne, Switzerland; 3 Department of Radiology, Lausanne University Hospital, Rue du Bugnon 46, 1011 Lausanne, Switzerland; 4 Division of Cardiology, Department of Advanced Biomedical Sciences, University of Naples Federico II, Naples, Italy

**Keywords:** Computed tomography angiography, Coronary artery disease, Transcatheter aortic valve implantation, Aortic valve stenosis

## Abstract

**Background:**

Computed tomography angiography (CTA) is used to plan TAVI procedures. We investigated the performance of pre-TAVI CTA for excluding coronary artery disease (CAD).

**Methods:**

In total 127 patients were included. CTA images were analyzed for the presence of ≥ 50% (significant CAD) and ≥ 70% (severe CAD) diameter stenoses in proximal coronary arteries. Results were compared with invasive coronary angiography (ICA) at vessel and patient levels. Primary endpoint was the negative predictive value (NPV) of CTA for the presence of CAD.

**Results:**

A total of 342 vessels were analyzable. NPV of CTA was 97.5% for significant CAD and 96.3% for severe CAD. Positive predictive value and accuracy were 44.8% and 87.1% for significant CAD and 56.3% and 94.4% for severe CAD. At patient level, NPV for significant CAD was 88.6%.

**Conclusion:**

Pre-TAVI CTA shows good performance for ruling out CAD and could be used as a gatekeeper for ICA in selected patients.

**Graphical abstract:**

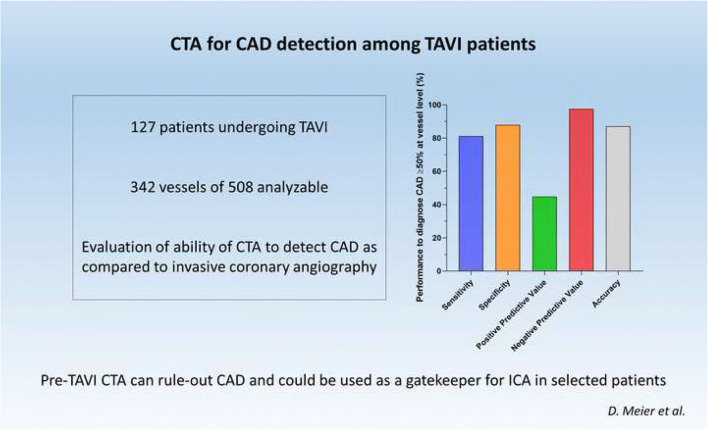

**Supplementary Information:**

The online version contains supplementary material available at 10.1007/s12265-021-10099-8.

## Background

Aortic valve stenosis (AS) is the most frequent form of valvular heart disease in Western countries [1]. In population-based studies, the prevalence of at least moderate AS is estimated at 2.8% among adults aged > 75 years, a percentage that is expected to rise in an aging population [2]. Given the rapidly expanding pool of evidence regarding the safety of transcatheter aortic valve implantation (TAVI) [3–5], the number of patients who could benefit from TAVI is increasing. Among them, an important proportion suffers from multiples comorbidities, including coronary artery disease (CAD). The reported prevalence of CAD among patients undergoing TAVI lies between 34 and 75%, with this significant variability arising from disparities between studies with regard to the definition of CAD and the assessment of coronary stenosis by angiography [6]. Given the high prevalence of CAD in this population, assessment using invasive coronary angiography (ICA) forms part of the routine workup before TAVI [7].

This workup also includes computed tomography angiography (CTA) to evaluate the dimensions of the aorta, the aortic annulus, and the peripheral vessels. CTA in this context is different (and thought to be less precise) than coronary CTA, which is more constraining due to requirement for heart rate control (< 65 bpm) and vessel dilatation with nitroglycerin [8, 9].

CTA has gained prominence in recent years and now occupies a place in the latest European guidelines on chronic coronary syndromes, where its use is recommended among patients with a lower pre-test probability of CAD [10]. However, among patients with a high pre-test probability of CAD, current guidelines (including those on valvular disease management) still recommend ICA as the first-line investigation [11, 12].

It is unclear whether CTA performed during the TAVI workup is accurate enough to exclude significant coronary artery disease, thus avoiding unnecessary ICA. This would be of particular interest among older, frailer patients with comorbidities such as chronic renal failure, where the risks of ICA are more significant. Moreover, the indication for TAVI is now expanding toward lower risk and younger patients with an expected lower burden of CAD and potentially higher image quality. Thus, an increasing role of CTA can be expected. Numerous studies have attempted to address this question with varying results [13–17]. However, these studies are limited by the use of highly selected populations, notably with the exclusion of higher-risk patients such as those with significant renal impairment [13, 16, 17].

In the current study, we aimed to evaluate the diagnostic performance of CTA in the diagnosis of CAD among an unselected cohort of patients selected for TAVI.

## Methods

### Study Design and Population

This is a retrospective study aiming to evaluate the performance of non-coronary dedicated pre-TAVI CTA in the diagnosis of CAD in patients undergoing transcatheter aortic valve implantation in our institution from the 1 of June 2013 to the 31 of December 2017.

The patients included in the present study are a subgroup of the SWISS TAVI registry, which is a national, multi-center, prospective cohort study collecting clinical characteristics of patients undergoing transcatheter aortic valve implantation (TAVI) in Switzerland. The study was approved by the ethic committee, and all patients gave their written informed consent.

All patients evaluated for potential TAVI in our center were eligible, while the only two exclusion criteria were previous CABG and unavailability of CTA images.

### CTA Protocol and Analysis

CT scans were performed using a 64-row detector CT scanner (LightSpeed VCT; GE Healthcare) with retrospective gating until the 31 of December 2013 and using a 256-row system (Revolution CT; GE Healthcare) with prospective gating thereafter. Image acquisition was also performed for patients in atrial fibrillation and with a heart rate of up to 128 bpm. Intravenous injection of 80–100 mL iohexol (350 mg I/mL, Accupaque® 350, GE Healthcare) into an antecubital vein was performed using a power injector at a flow rate of 5 mL/s, followed by a saline chaser. The bolus tracking technique with manual triggering was applied to start the acquisition. The acquisition parameters were as follows: rotation speed, 0.35 s (64-row system) and 0.28 s (256-row system); tube potential, 100 or 120 kVp depending on body mass index; and tube current, 400–600 mA. Neither heart rate control nor vasodilatation (nitroglycerin) was used prior to the procedure as CTA was not coronary dedicated. The image reconstruction parameters were as follows: section thickness, 0.625 mm; kernel, soft tissue; algorithm, adaptive statistical image reconstruction 50% (64-row system) and adaptive statistical image reconstruction-V 50% (256-row system); display field-of-view, 28 cm; and cardiac phase, end-systolic. In addition to the 28 cm field-of-view, a dedicated cardiac reconstruction with a 20 cm field-of-view was provided for coronary artery analysis. CTA images were retrospectively read by two experienced radiologists, who were blinded from ICA images and reports. To determine the inter-observer variability using the κ of Cohen test, 15% of images were randomly selected and read by both radiologists separately. The four main coronary arteries were evaluated (right coronary artery, left main artery, left circumflex artery, and left anterior descending artery). The quality of each vessel was rated using a 3-point Likert scale in terms of delineation between lumen and wall of the artery, and filling with contrast material: optimal (no artifacts, strong attenuation of vessel lumen), suboptimal (moderate artifacts, low contrast, but acceptable for routine clinical diagnosis), or unanalyzable (severe artifacts or insufficient contrast impairing accurate evaluation). CAD analysis was undertaken on all vessels ≥ 2.5 mm whose quality was rated as optimal or suboptimal. The following classification for CAD was applied: no significant CAD (0–49% lumen diameter reduction), moderate CAD (50–69% lumen diameter reduction), and severe CAD (70–99% lumen diameter reduction or occlusion). If there was more than one stenosis on a vessel, only the most severe lesion was taken into account. The Agatston calcium score was also calculated in the coronary arteries.

### ICA Protocol and Analysis

All patients underwent ICA with standard views. The ICA images were retrospectively analyzed by an experienced interventional cardiologist blinded to CTA images and results. A CAD visual analysis was performed in each vessel ≥ 2.5 mm using the same classification as for the CTA analysis. Significant CAD was defined as ≥ 50% lumen diameter reduction, and severe CAD was defined as ≥ 70% lumen diameter reduction.

### Endpoints

The primary endpoint was the performance of CTA as a tool for excluding the presence of significant CAD. This was measured in terms of negative predictive value (NPV), using ICA as gold standard. Secondary endpoints included sensitivity, specificity, positive predictive value (PPV), and accuracy (ACC) of CTA.

### Subgroup Analysis

Subgroup analyses based on CTA quality were performed at vessel level with a subgroup with vessel images of optimal quality and another subgroup with vessel images of suboptimal quality.

### Assessment of Predictors of Erroneous CTA Interpretation and Unanalyzable CTA

To assess whether some clinical characteristics where associated with image quality, we sought to determine potential predictors of erroneous CTA image interpretation and the acquisition of unanalyzable CTA images. To this end, a multivariate binary logistical regression was performed including: age, gender, body mass index, type of CT used, history of previous PCI, diabetes, renal failure, dyslipidemia, hypertension, and chronic obstructive pulmonary disease.

### Statistical Analysis

Statistical analysis was carried out using SPSS 26.0 software (SPSS Inc., Chicago, IL). Continuous variables were expressed as means ± standard deviation or medians (P25; P75), while categorical variables were expressed as frequencies (percentage). The inter-observer variability was calculated using the *κ* of Cohen test, for the quality assessment and for the diagnosis of significant CAD at vessel level. A *κ* of 0.51 was found for the quality assessment, which corresponds to a moderate inter-observer agreement. For the diagnosis of significant CAD at vessel level, *κ* was calculated at 0.61, which corresponds to a good inter-observer agreement. Both agreements were significant with a *p* value < 0.05.

The analysis was conducted at patient level and at vessel level. For the patient-level analysis, a patient was considered positive for CAD when at least one vessel showed CAD (defined as previously with the two different cut-offs of ≥ 50% lumen diameter reduction for significant CAD and ≥ 70% lumen diameter reduction for severe CAD). The patient-level analysis was only performed on 74 patients whose 4 arteries had an analyzable quality on CTA. For the prediction of incorrect CTA image interpretation and the acquisition of unanalyzable CTA images, we performed a patient-level binary logistic regression, including, age, gender, body mass index, presence of diabetes, dyslipidemia, COPD, hypertension, GFR < 45 mL/min, type of CT used (64- vs 256-row detector), and history of previous PCI. Role of coronary calcification in the occurrence of unanalyzable images and error in CTA interpretation was also assessed using binary logistic regression.

## Results

### Patients Characteristics

**Fig. 1:**
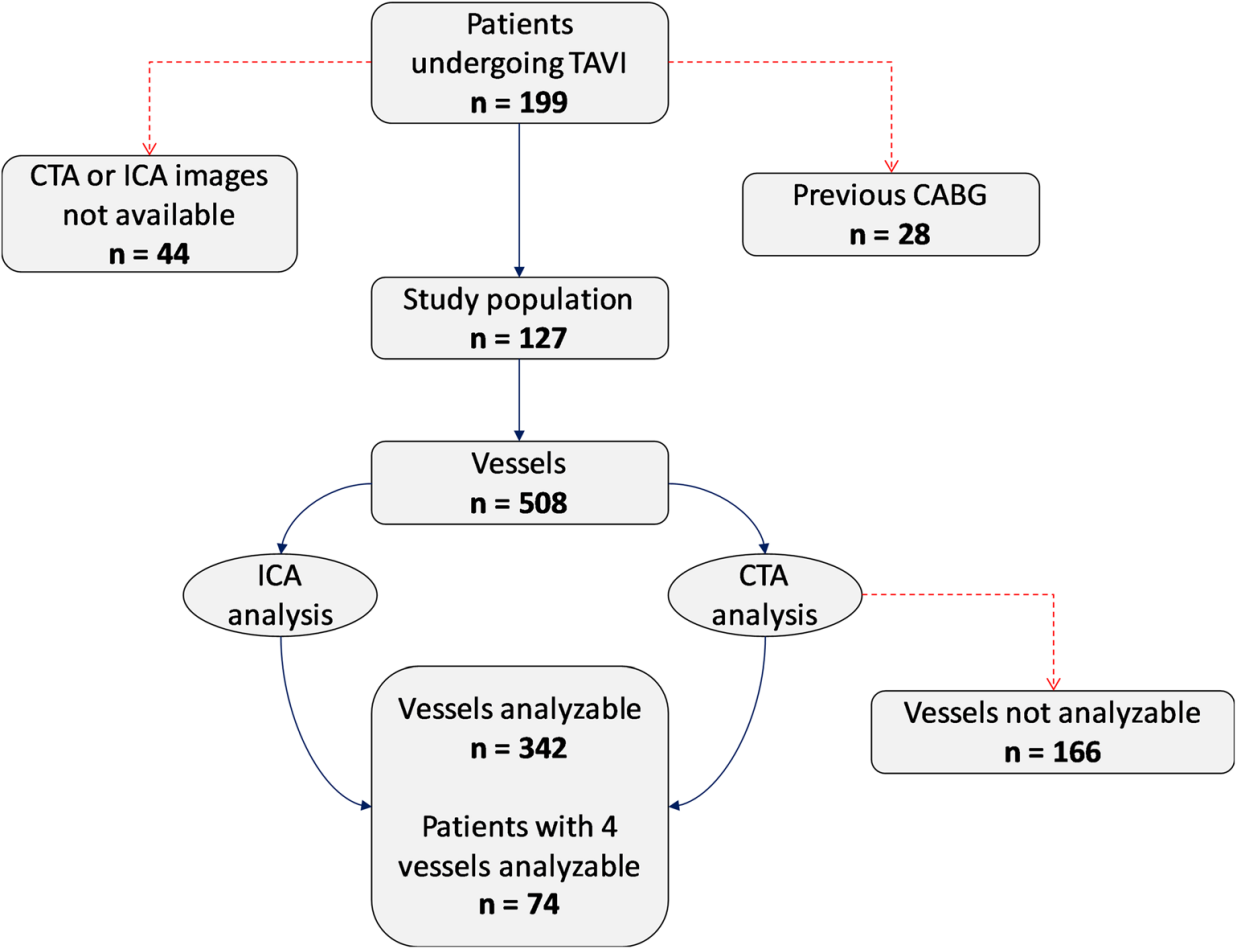
Flowchart of the study design. CABG: coronary artery bypass graft; CAD: coronary artery disease; ICA: invasive coronary angiography; CTA: computed tomography angiography

In total, 199 consecutive patients fulfilled the initial inclusion criteria, but 44 patients (22%) were excluded due to the unavailability of CTA or ICA images, as a result of imaging being performed in a different center. In addition, 28 patients with previous CABG were also excluded. The final study population includes 127 patients (Fig. 1).

Patient baseline characteristics are summarized in Table 1. Mean age was 82.3 years ± 7.3. Dyslipidemia and arterial hypertension were present among the majority of the patients with a prevalence of 54.3% and 77.2%, respectively. The median estimated glomerular filtration rate was 43 mL/min/1.73 m^2^ [36; 58], and mean heart rate during images acquisition was 73 ± 18.6. There were 21 patients with a history of percutaneous coronary intervention, and the median mortality risk after a cardiac intervention estimated with the EuroSCORE II was 3.7% [2.3; 5.4].

**Table 1:** Baseline characteristics of patients

Baseline characteristic *(n* = 127)	Value
Age (years)	82.3 ± 7.3
Male	49 (38.6%)
BMI (kg/m2)	26.5 ± 5.1
Dyslipidemia	69 (54.3%)
Arterial hypertension	98 (77.2%)
Diabetes mellitus	36 (28.3%)
COPD	15 (11.8%)
History of PCI	21 (16.5%)
eGFR (mL/min/1.73 m)	43 (36; 58)
EuroSCORE II (%)	3.7 (2.3; 5.4)
Heart rate during CTA acquisition	73 ± 18.6
Agatston calcium score	703 (195; 1665)
Coronary artery disease	49 (38.6%)
*1 vessel disease*	33 (26%)
*2 vessels disease*	14 (10.9%)
*3 vessels disease*	2 (1.6%)

*BMI* body mass index, *COPD* chronic obstructive pulmonary disease, *eGFR* estimated glomerular filtration rate, *PCI* percutaneous coronary intervention. Age BMI and heart rate are expressed in mean ± standard deviation while eGFR, EuroSCORE II, and Agatston calcium score are expressed in median (P25; P75)

### CTA Quality

A total of 508 vessels were evaluated (4 vessels in 127 patients). Among them, 166 vessels (32.7%) in 33 patients were considered as unanalyzable and were therefore excluded from the analysis. Therefore, the final data set consisted of 342 vessels in 94 patients with image quality classified as optimal in 141 (27.8%) and suboptimal in 201 (39.6%). The left main artery (LM) had the highest rate of optimal quality images (49.6%). On patient-level analysis, among the 127 patients included, 74 (58.3%) had 4 analyzable coronary vessels, 9 (7.1%) had 3 analyzable vessels, 8 (6.3%) had 2 analyzable vessels, and 3 (2.4%) patients had only one analyzable vessel. Thirty-three (25.8%) patients had all four arteries classed as unanalyzable. CTA image quality results are presented in detail in Table 2**.** The median Agatston calcium score was 703 [195;1665] but was only calculated among 83 (65.4%) patients. Among the remaining patients, calcium scores were not calculable due to either the presence of significant noise in the images (21 patients, 16.5%) or because the dedicated native sequence was not available (23 patients, 18.1%).

**Table 2:** CTA images quality results

Vessel	Quality (*n* (%))		
	Optimal	Suboptimal	Unanalyzable
RCA (*n* = 127)	29 (22.8%)	52 (40.9%)	46 (36.2%)
LM (*n* = 127)	63 (49.6%)	29 (22.7%)	35 (27.3%)
LCX (*n* = 127)	24 (18.8%)	59 (22.8%)	44 (34.6%)
LAD (*n* = 127)	25 (19.7%)	61 (48%)	41 (32.3%)
All vessels (*n* = 508)	141 (27.8%)	201 (39.6%)	166 (32.7%)

*LAD* left anterior descending artery, *LCX* left circumflex artery, *LM* left main artery, *RCA* right coronary artery

### ICA Results

A total of 508 vessels were analyzed, and a significant stenosis (≥ 50%) was found in 67 (13.2%) of them. On patient-level analysis, ICA revealed significant CAD (≥ 50%) in 49 patients (38.6%) and severe CAD (≥ 70%) in 29 patients (22.8%).

### CTA Performance in the Whole Cohort at a Vessel Level

The primary endpoint (performance of CTA in excluding significant CAD (≥ 50%)) was evaluated on 342 vessels. The NPV was 97.5%. Secondary endpoints were determined on the same number of vessels and were as follows: sensitivity, 81.1%; specificity, 87.9%; PPV, 44.8%; and ACC, 87.1%. Detailed analysis for each vessel (RCA, LCX, LAD, and LM) is presented in Table 3. For the exclusion of severe CAD (≥ 70%), the NPV was at 96.3%. Sensitivity is at 42.8%, specificity at 97.8%, PPV at 56.3%, and ACC at 94.4% (Table 4).

**Table 3:** CTA performance to diagnose significant CAD (≥ 50% diameter reduction)

Vessel	N	TP (*n*)	TN (*n*)	FP (*n*)	FN (*n*)	Sensitivity (%)	Specificity (%)	PPV (%)	NPV (%)	Accuracy (%)
RCA	81	9	67	5	0	100	93.1	64.3	100	93.8
LM	92	1	86	5	0	100	94.5	16.7	100	94.6
LCX	83	3	67	11	2	60	85.9	26.7	97.1	84.3
LAD	86	17	48	16	5	73.9	75	51.5	90.6	74.7
All vessels	342	30	268	37	7	81.1	87.9	44.8	97.5	87.1

*FN* false negative, *FP* false positive, *LAD* left anterior descending artery, *LCX* left circumflex artery, *LM* left main artery, number *NPV* negative predictive value, *PPV* positive predictive value, *RCA* right coronary artery, *TN* true negative, *TP* true positive

**Table 4:** CTA performance to diagnose severe CAD (≥ 70% diameter reduction)

Vessel	*N*	TP (*n*)	TN (*n*)	FP (*n*)	FN (*n*)	Sensitivity (%)	Specificity (%)	PPV (%)	NPV (%)	Accuracy (%)
RCA	81	4	74	2	1	80	97.4	66.7	98.7	96.3
LM^1^	92	0	91	0	1	-	-	-	98.9	-
LCX	83	1	77	3	2	33.3	96.3	25	97.5	94
LAD	86	4	72	2	8	33.3	97.3	66.7	90	88.4
All vessels	342	9	314	7	12	42.8	97.8	56.3	96.3	94.4

*FN* false negative, *FP* false positive, *LAD* left anterior descending artery, *LCX* left circumflex artery, *LM* left main artery, number *NPV* negative predictive value, *PPV* positive predictive value, *RCA* right coronary artery, *TN* true negative, *TP* true positive

^1^Sensitivity, specificity, positive predictive value, and accuracy could not be calculated for the left main artery as there was no severe stenosis on this artery

### CTA Performance in Subgroups at Vessel Level

A subgroup analysis was conducted according to CTA image quality. CTA with optimal quality images performed slightly better than CTA with suboptimal quality images, with a NPV of 99.2 versus 95.9%, respectively, with regard to the exclusion of significant CAD, and 98.5% versus 94.7%, respectively, with regard to the exclusion of severe CAD. Similarly, accuracy was slightly better for CTA with optimal quality images (91.4% vs 84.1% for significant CAD and 96.4% vs 93% for severe CAD) (Supplementary Tables 1, 2 and Supplementary Fig. 1**)**.

### CTA Performance at Patient Level

Using the 50% cut-off, CTA had a negative predictive value of 88.6% and a positive predictive value of 56.4 %. For the 70% cut-off, negative and positive predictive values were, respectively, 89.1% and 70%.

### Predictors of Error in CTA Interpretation and Unanalyzable CTA

Using the 50% cut-off, only the body mass index was negatively associated with incorrect CTA interpretation (OR 0.85, [0.75; 0.99]). Of note, chronic renal failure with a GFR < 45 mL/min was also negatively associated with incorrect CTA interpretation although the association did not reach statistical significance (OR 0.52, [0.17; 1.60], *p* = 0.046) (Supplementary Table 3). Regarding the occurrence of unanalyzable CTA images, the use of a 64-row detector for images acquisition was the only parameter significantly associated with the acquisition of unanalyzable images (OR 8.80, [1.88; 41.13], *p* = 0.006) (Supplementary Table 4). Total amount of coronary calcifications was neither associated with error in CTA interpretation (OR 1.04, [0.99; 1.01], *p* = 0.135 for an increase of 100 points in calcium score) nor with the acquisition of unanalyzable images (OR 1.05, [0.99; 1.11], *p* = 0.098 for an increase of 100 points in calcium score).

## Discussion

The main findings of the present study can be summarized as follows:

1. CTA has a very good negative predictive value for the presence of CAD in vessels ≥ 2.5 mm among TAVI patients, permitting the exclusion of significant CAD even in the absence of a dedicated coronary imaging protocol.
2. CTA has a high false-positive rate for obstructive CAD.
3. Clinical characteristics of patients cannot be reliably used to predict the occurrence of error during CTA interpretation or the acquisition of unanalyzable images.

A recent meta-analysis from Van Den Boogert et al. that compiled data from 7 single-center studies analyzed the performance of pre-TAVI CTA in the diagnosis of significant CAD. At patient level, it identified values for sensitivity, specificity, PPV, and NPV of 95%, 65%, 71%, and 94%, respectively [18]. However, 6 out of these 7 studies included patients with prior CABG. The only study that did exclude CABG patients was Rossi and al [19]. This study included 140 patients and showed similar results to ours in terms of sensitivity, specificity, positive, and negative predictive values. Moreover, many of the studies did not enroll patients with significantly reduced renal function, thus potentially limiting their applicability to a real-world population [13, 16, 17]. Finally, heart rate control was not used during image acquisition, and mean heart rate was 73 bpm, which is slightly higher than reported in the studies included in the meta-analysis. This is particularly relevant in this fragile population at higher risk of side effects related to the drugs used for rate control.

Our results suggest that the NPV of CTA for the presence of CAD is good among patients selected for TAVI, with an NPV of almost 90% for significant and severe CAD on a patient level. This was also true for patients with significantly reduced renal function.

However, CTA tends to over-diagnose obstructive CAD, with a positive predictive value of only 56.4% for significant CAD, implying a false-positive rate of more than 40%.

These two considerations suggest that CTA could be used as a gatekeeper for ICA in the TAVI workup. Indeed, ICA can probably be safely deferred if all 4 vessels are analyzable and found to be free of obstructive CAD. In the present cohort, almost 60% of patients had 4 vessels analyzable, rendering a patient-level analysis possible. Among these patients, potentially obstructive CAD could be excluded in almost 50% with a relatively high degree of certainty. This means that among the whole cohort, ICA could have been avoided in almost one third of patients. This is an important finding as the median GFR was 43 mL/min suggesting that a contrast-sparing strategy could be of high value in this fragile population. This also has potential benefits with regard to the reduction of other iatrogenic complications as well as costs related to ICA. These results are especially interesting as they come from images initially focused on vascular access rather than the coronary tree with no special preparation such as aggressive heart rate control and vasodilation. Moreover, a small proportion (22.8%) of patients was found to have severe CAD on ICA: This suggests that using prospective dedicated imaging protocols and latest generation CT scanners, the proportion of patients with 4 vessels analyzable and in whom ICA could be avoided is susceptible to be even higher than in the present study.

Interestingly, the subgroup analysis, showed a higher sensitivity and positive predictive value in the suboptimal quality group, explained by the fact that the prevalence of CAD was higher in suboptimal quality vessels than in those of optimal quality as higher levels of calcification are partly responsible for the degradation of image quality. Finally, to our knowledge, this is the first study reporting data on factors predictive of error in CTA interpretation and the occurrence of unanalyzable images. Here, we show that no single clinical characteristic can be reliably used to successfully predict these variables.

## Limitations

The main limitation of the present study was that the number of unanalyzable vessels was higher than in the previously cited meta-analysis [18]. Indeed, 32.7% of vessels were unanalyzable in our study, which is likely due, in part, to the retrospective setting and the unselected population. Nevertheless, almost 60% of patients had 4 vessels analyzable. It must also be taken into account that these results were derived from a retrospective cohort, with no special focus on CAD during initial image acquisition, indicating that the performance could probably be higher if sequences were optimized to assess the coronary tree.

Another debatable aspect is to determine whether a 90% level of certainty is considered high enough to avoid further testing. Regarding this point, it must be stated that the performance of CTA was dependent on vessel size. For example, the NPV for LM stenosis was 100%. Thus, CTA appears to perform well in the detection of proximal lesions with a high prognostic impact, while the lower mean performance comes from a more difficult analysis of distal segments in which significant CAD is more difficult to diagnosis and could be missed by CTA. This seems relevant as TAVI patients are generally older patients, in whom the identification of potentially life-threatening lesions in the short term holds particular importance.

Finally, we noted only a moderate level of inter-observer agreement with regard to the interpretation of the CTA images. This likely reflects the degree of subjectivity associated with certain aspects of image interpretation.

## Conclusion

In conclusion, general pre-TAVI CTA performs well as a tool for ruling out significant and severe CAD and could be used as a gatekeeper for ICA if image quality is acceptable. This is especially true among patients with impaired renal function.

## Clinical Relevance

The present study investigated the performance of pre-TAVI CTA in excluding CAD. The negative predictive value of CTA was 97.5% for the diagnosis of significant CAD defined as stenosis ≥ 50% on ICA. Thus, these data suggest that pre-TAVI CTA could be used as a gatekeeper for ICA in selected patients. This is particularly relevant as TAVI patients are often frail and at high-risk of ICA-related complications.

## Supplementary Information

Supplementary Fig. 1 Comparison of the performance of CTA to diagnose CAD between optimal and suboptimal quality vessels (PNG 1066 kb)

High resolution image (TIF 466 kb)

ESM 1 (DOCX 30.4 kb)

## Abbreviations

ACC: Accuracy
AS: Aortic stenosis
TAVI/R: Transcatheter aortic valve implantation/replacement
CAD: Coronary artery disease
CTA: Computed tomography angiography
ICA: Invasive coronary angiography
CABG: Coronary artery bypass graft
NPV: Negative predictive value
PPV: Positive predictive value
RCA: Right coronary artery
LCX: Left circumflex
LAD: Left anterior descending
LM: Left main
