# Supplementary figures and images for: Computed Tomography Angiography for the Diagnosis of Coronary Artery Disease Among Patients Undergoing Transcatheter Aortic Valve Implantation
Source: J Cardiovasc Transl Res. 2021 Feb 4;14(5):894–901. doi: 10.1007/s12265-021-10099-8 (PMC8575747; doi:10.1007/s12265-021-10099-8)

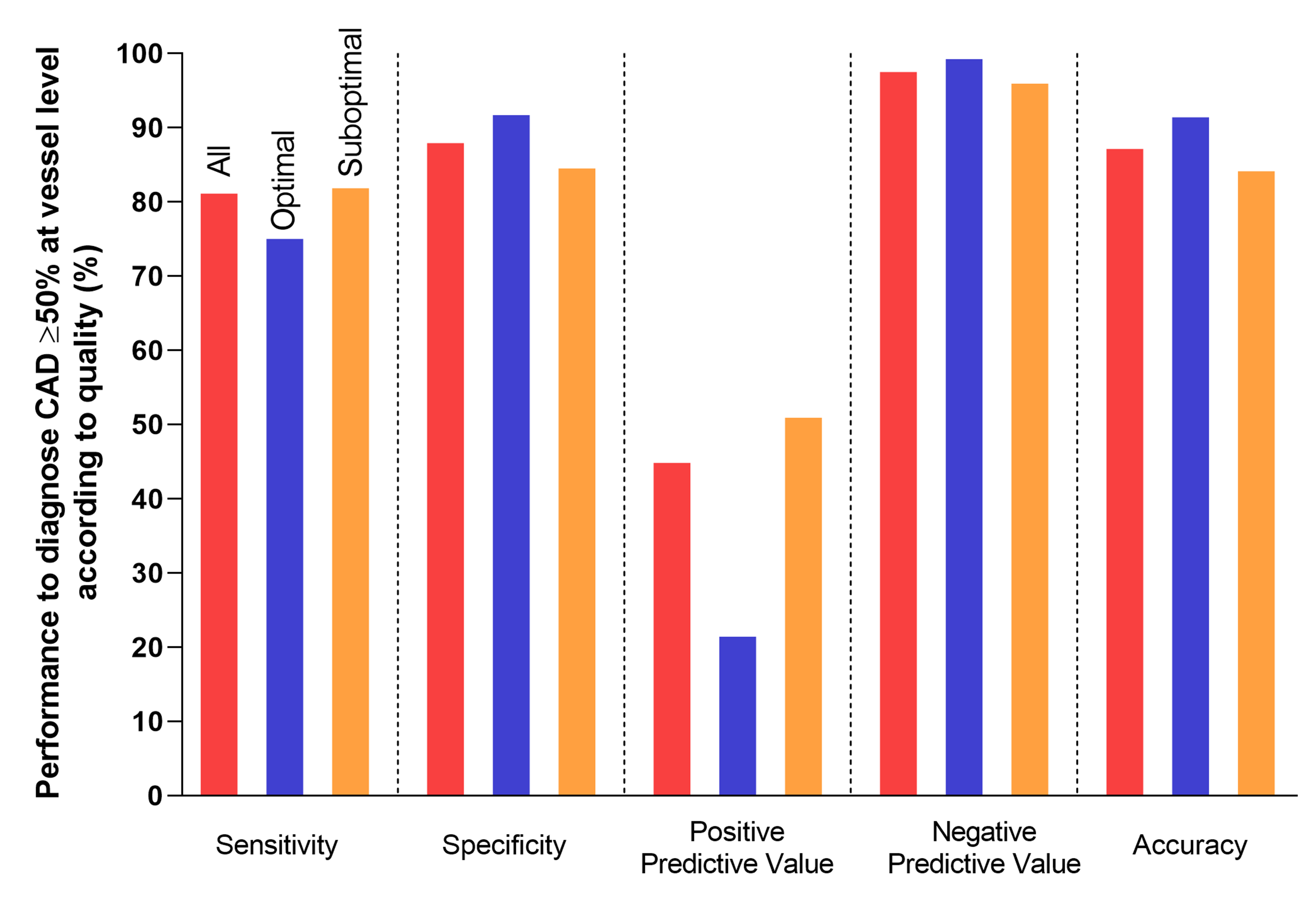

Supplement: Supplementary file 1 — Comparison of the performance of CTA to diagnose CAD between optimal and suboptimal quality vessels (PNG 1066 kb) [file 12265_2021_10099_Fig2_ESM.png]

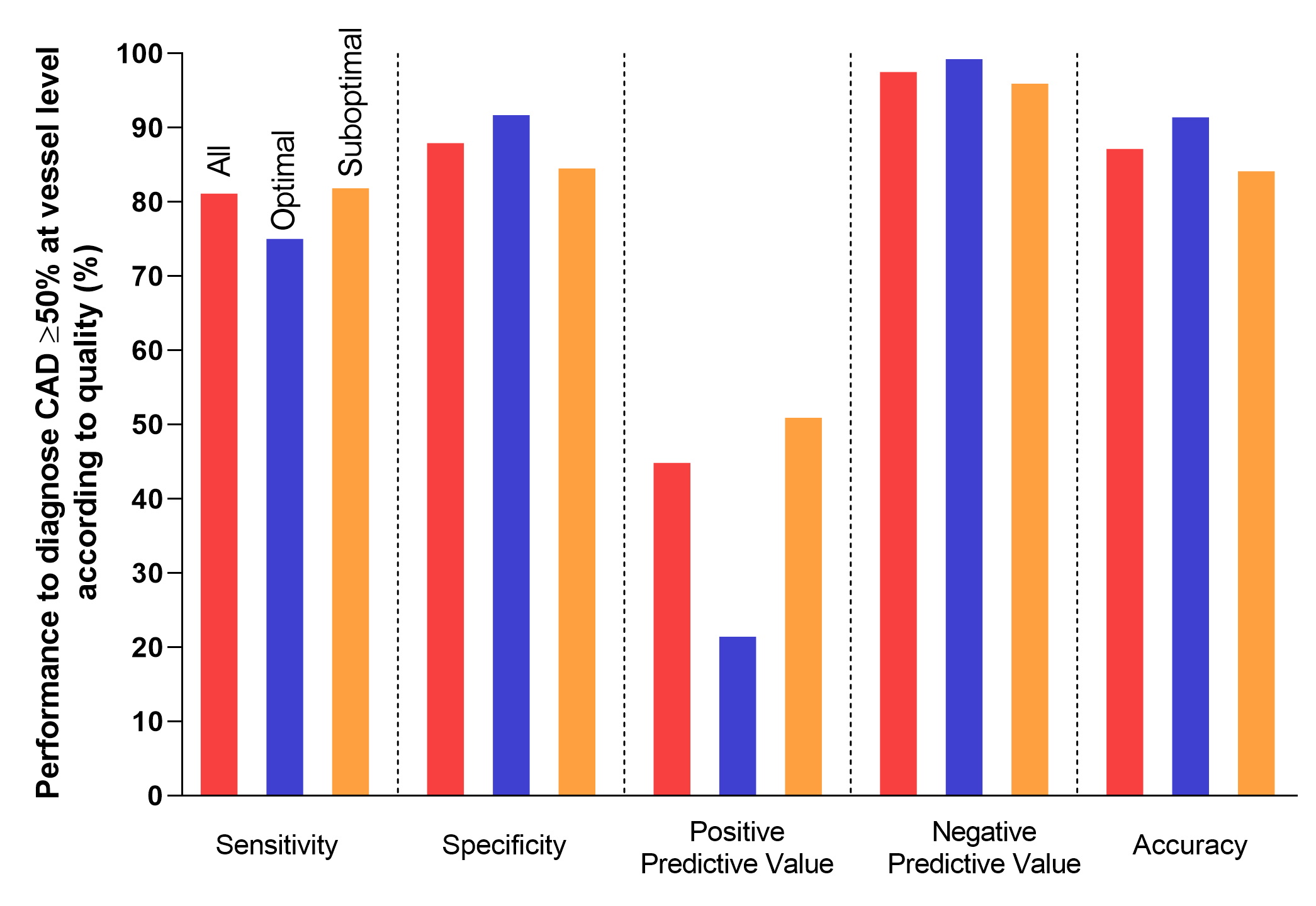

Supplement: Supplementary file 2 — High resolution image (TIF 466 kb) [file 12265_2021_10099_MOESM1_ESM.tif]
