# Supplementary material for: Computed Tomography Angiography for the Diagnosis of Coronary Artery Disease Among Patients Undergoing Transcatheter Aortic Valve Implantation
Source: J Cardiovasc Transl Res. 2021 Feb 4;14(5):894–901. doi: 10.1007/s12265-021-10099-8 (PMC8575747; doi:10.1007/s12265-021-10099-8)
Supplement: Supplementary file 3 — (DOCX 30.4 kb) [file 12265_2021_10099_MOESM2_ESM.docx]

**SUPPLEMENTARY TABLES**

**Supplementary table 1: Subgroup analysis to diagnose significant CAD (≥50%) according to the quality of the vessels**

| *Quality* | *N* | *TP (n)* | *TN (n)* | *FP (n)* | *FN (n)* | *Sensitivity (%)* | *Specificity (%)* | *VPP (%)* | *VPN (%)* | *Accuracy (%)* |
| --- | --- | --- | --- | --- | --- | --- | --- | --- | --- | --- |
| Optimal | 141 | 3 | 126 | 11 | 1 | 75 | 91.7 | 21.4 | 99.2 | 91.4 |
| Suboptimal | 201 | 27 | 142 | 26 | 6 | 81.8 | 84.5 | 50.9 | 95.9 | 84.1 |
| Table 5: FN false negative, FP false positive, LAD left anterior descending artery, LCX left circumflex artery, LM left main artery, number NPV negative predictive value, PPV positive predictive value, RCA right coronary artery, TN true negative, TP true positive. | | | | | | | | | | |

Supplementary table 1: Table 5: FN false negative, FP false positive, LAD left anterior descending artery, LCX left circumflex artery, LM left main artery, number NPV negative predictive value, PPV positive predictive value, RCA right coronary artery, TN true negative, TP true positive.

**Supplementary table 2: Subgroup analysis to diagnose severe CAD (≥70%) according to the quality of the vessels**

| *Quality* | *N* | *TP (n)* | *TN (n)* | *FP (n)* | *FN (n)* | *Sensitivity (%)* | *Specificity (%)* | *VPP (%)* | *VPN (%)* | *Accuracy (%)* |
| --- | --- | --- | --- | --- | --- | --- | --- | --- | --- | --- |
| Optimal | 141 | 1 | 135 | 3 | 2 | 33.3 | 97.8 | 25 | 98.5 | 96.4 |
| Suboptimal | 201 | 8 | 179 | 4 | 10 | 44.4 | 97.8 | 66.7 | 94.7 | 93 |

Supplementary table 2: Table 5: FN false negative, FP false positive, LAD left anterior descending artery, LCX left circumflex artery, LM left main artery, number NPV negative predictive value, PPV positive predictive value, RCA right coronary artery, TN true negative, TP true positive.

**Supplementary table 3: Predictors of error in CTA interpretation using the 50% cut-off**

| Determinant | OR (95% CI) | p-value |
| --- | --- | --- |
| Previous PCI | 1.805 [0.454;7.168] | 0.401 |
| Diabetes | 0.795 [0.229;2.753] | 0.717 |
| Body Mass Index | 0.867 [0.753;0.998] | **0.046** |
| Gender | 1.889 [0.620;5.752] | 0.263 |
| Age | 1.053 [0.951;1.165] | 0.322 |
| Renal failure | 0.442 [0.140;1.394] | 0.164 |
| Dyslipidemia | 1.907 [0.617;5.896] | 0.262 |
| Hypertension | 1.957 [0.519;7.380] | 0.321 |
| COPD | 0.423 [0.070;2.245] | 0.348 |
| Use of a 64-row detector | 7.597 [0.679;84.977] | 0.100 |

*Supplementary table 3: COPD: Chronic Obstructive Pulmonary disease; PCI: Percutaneous Coronary Intervention*

**Supplementary table 4: Predictors of unanalyzable CTA**

| Determinant | OR (95% CI) | p-value |
| --- | --- | --- |
| Previous PCI | 0.835 [0.266;2.616] | 0.757 |
| Diabetes | 0.754 [0.267;2.126] | 0.593 |
| Body Mass Index (kg/m^2)^ | 1.053 [0.956;1.160] | 0.297 |
| Male sex | 1.713 [0.681;4.308] | 0.252 |
| Age (per increase of 1 year) | 0.956 [0.895;1.022] | 0.185 |
| Renal failure | 1.932 [0.718;5.199] | 0.193 |
| Dyslipidemia | 1.712 [0.662;4.308] | 0.268 |
| Hypertension | 2.977 [0.775;11.438] | 0.112 |
| COPD | 0.263 [0.058;1.182] | 0.081 |
| Use of a 64-row detector | 8.796 [1.881;41.134] | **0.006** |

*Supplementary table 4: COPD: Chronic Obstructive Pulmonary Disease; PCI: Percutaneous Coronary Intervention*
